# Herbal Medicines against Hydatid Disease: A Systematic Review (2000–2021)

**DOI:** 10.3390/life12050676

**Published:** 2022-05-02

**Authors:** Mughees Aizaz Alvi, Sadiq Khan, Rana Muhammad Athar Ali, Warda Qamar, Muhammad Saqib, Noman Yousaf Faridi, Li Li, Bao-Quan Fu, Hong-Bin Yan, Wan-Zhong Jia

**Affiliations:** 1 State Key Laboratory of Veterinary Etiological Biology, National Professional Laboratory for Animal Echinococcosis, Key Laboratory of Veterinary Parasitology of Gansu Province, Lanzhou Veterinary Research Institute, Chinese Academy of Agricultural Sciences, Lanzhou 730046, China; mugheesaizazalvi@gmail.com (M.A.A.); lili03@caas.cn (L.L.); fubaoquan@caas.cn (B.-Q.F.); 2 Department of Clinical Medicine and Surgery, University of Agriculture, Faisalabad 38000, Pakistan; sadiq2859@gmail.com (S.K.); athar4545@gmail.com (R.M.A.A.); drsaqib_vet@hotmail.com (M.S.); 3 Department of Parasitology, University of Agriculture, Faisalabad 38000, Pakistan; wardaqamar17@gmail.com; 4 Livestock and Dairy Development Department, Government of Punjab, Lahore 54000, Pakistan; nomanfaridi786@gmail.com; 5 Jiangsu Co-Innovation Center for Prevention and Control of Important Animal Infectious Disease, Yangzhou 225009, China

**Keywords:** medicinal plants, *Echinococcus granulosus*, efficacy, in vitro, in vivo

## Abstract

Echinococcosis is a serious public health issue that affects people and livestock all over the world. Many synthetic and natural products have been examined in vitro and in vivo on *Echinococcus* species but only a few are used clinically, however, they may cause some complications and side effects. To overcome these limitations, new horizons of herbal drugs to cure echinococcosis are opening with every passing day. To summarize the developments during the last 21 years, we conducted this review of the literature to identify medicinal herbs utilized throughout the world that have anti-*Echinococcus* activity. From 2000 to 2021, data were carefully obtained from four English databases: Science Direct, PubMed, Scopus, and OpenGrey. Botanical name, extraction technique, extract quantities, efficacy, duration of treatment, year of publication, and half-maximal inhibitory concentration (IC_50_) values were all well noted. Ninety-one published papers, with 78 in vitro and 15 in vivo, fulfilled our selection criteria. Fifty-eight different plant species were thoroughly tested against *Echinococcus* *granulosus*. *Zataria multiflora*, *Nigella sativa*, *Berberis vulgaris*, *Zingiber officinale* (ginger), and *Allium sativum* were the most often utilized anti-*Echinococcus* herbs and the leaves of the herbs were extensively used. The pooled value of IC_50_ was 61 (95% CI 60–61.9) according to the random effect model and a large degree of diversity among studies was observed. The current systematic study described the medicinal plants with anti-*Echinococcus* activity, which could be investigated in future experimental and clinical studies to identify their in vivo efficacy, lethal effects, and mechanisms of action.

## 1. Introduction

Helminth parasite infections are classified as neglected tropical diseases (NTD) due to the lower research budget allocation [1]. Helminths are classified into two major phyla: Platyhelminthes including cestodes and trematodes, as well as nematodes [2]. Cystic echinococcosis (CE) is a chronic cestode zoonosis affecting humans as well as livestock animals [3]. It raises a significant public health issue in central Asia, China, South America, Europe, Australia, and Africa. The occurrence of this disease depends upon culture characteristics, and the health and economic status of community [4]. The definitive host (dog) harbors the mature *Echinococcus* parasite in the intestine, whose eggs release via feces and spread in environment, whereas cattle, sheep, goats, horses, and pigs are the intermediate hosts of this parasite, ingesting infectious mature eggs resulting in hydatid cysts in various parts of the body, especially on the liver and lungs [5,6]. CE infestation leads to economic losses in the form of condemnation of carcasses, decreased milk production, and fecundity, as well as increased weight loss and mortality [7].

The choice of treatment regimen depends upon the size, location, and number of hydatid cysts. At the present time, treatment options for cystic echinococcosis are active anthelmintics, surgery, and percutaneous aspiration [8,9]. However, these treatment strategies have major limitations. Many scolicidal agents such as povidone iodine, formalin, hydrogen peroxide, silver nitrate, cetrimide, and albendazole may be applied during surgery to the hydatid cysts to deactivate live protoscoleces and control the reoccurrence of infection but many complications have been reported [10,11]. Albendazole and mebendazole are the chemical drugs to cure hydatid cysts in human. These drugs are used in high doses for a long time to cure disease which ultimately results in hepatic toxicity and other adverse outcomes [12,13].

Herbal medicines are being used against a number of bacterial and parasitic diseases and are proven to be promising anti-parasitic agents [14,15,16]. With special reference to *Echinococcus* species, scientists are investigating to find new scolicidal agents with increased safety and efficacy.

Many synthetic and natural products have been examined in vitro and in vivo against *Echinococcus* species but only a few are used clinically, however, they may cause some complications and side effects [17]. Recently some herbal drugs to cure *Echinococcus* are being developed with low adverse effects, low cost, and high accessibility [18].

In this review, we collected the published literature on those plants which had active components for killing protoscoleces of *E. granulosus*. The main objective of this review was to explore ongoing trends in research investigating the scolicidal potential of herbal plants against *E. granulosus* and to fill the current knowledge gap in order to improve and clarify future research streams where more attention should be focused.

## 2. Material and Methods

### 2.1. Search Method

We conducted a systematic review of the literature on the treatment of *E. granulosus* using medicinal plants by adopting Table S1 PRISMA guidelines [19]. From 2000 to 2021, a comprehensive search was conducted across all scientific databases, including four English databases: Science Direct, Scopus, Pub Med, and OpenGrey. The searched topics were plant extract, herbal extract, medicinal plants, traditional medicine, and herbal medicine, whose effects on echinococcosis occurred when used alone or in combination. Some of the specific keywords used for retrieval of the published data from 2000–2021 included “scolicidal agents”, “natural scolicidal and protoscolicidal compounds”, “medicinal/herbal drugs used against *E*. *granulosus*”, “in vitro or in vivo activity of plants against *E*. *granulosus*”, “natural compounds against protoscoleces”, and “antihydatid agents”. Other pertinent issues, such as the *Echinococcus* parasite, were also looked at and added if the relevant results could be found.

### 2.2. Inclusion and Exclusion Criteria

**Figure 1:**
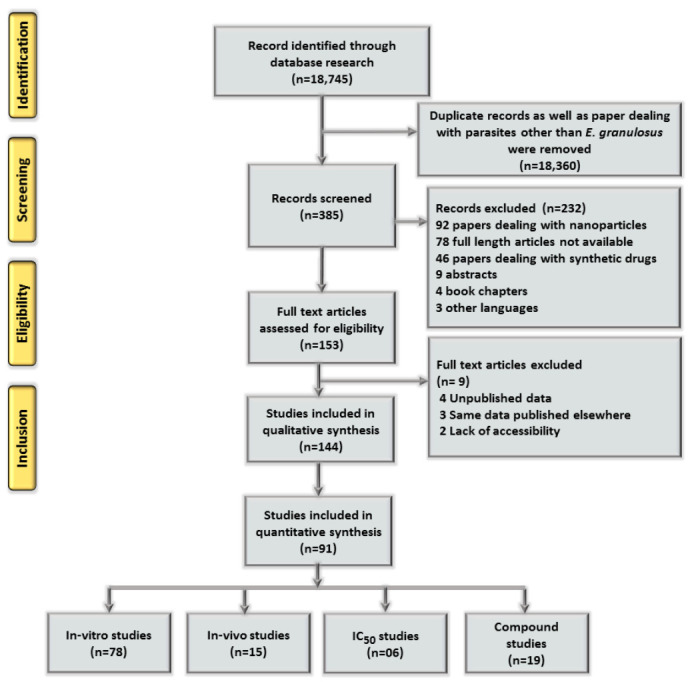
Flow chart indicating the screening process for the systematic review.

Reference screening was done based on the titles, and irrelevant and redundant references were deleted. Figure 1 shows a flow chart of article identification, screening, eligibility, and inclusion criteria. The last search was performed on 25 January 2022.

Studies with full text availability were considered for the current review. Studies which reported in vitro/in vivo scolicidal activity of plants against protoscoleces of *E*. *granulosus* were taken into account in this review. Studies reporting synthetic scolicidal agents, report related to activity of nanoparticles against *E*. *granulosus*, and studies describing agents used against other helminths did not fulfill the inclusion criteria and were excluded. Moreover, epidemiological and molecular investigations on *E*. *granulosus* did not meet the inclusion criteria.

A total of 18,745 publications were identified from the searches and reviewed. A total of 385 papers were retrieved after removal of duplicate papers, as well as articles dealing with parasites other than *E. granulosus*. Selected articles were screened and 232 papers dealing with nanoparticles, synthetic drugs, abstracts, book chapters and other languages were removed. Papers for which the full text was not available were also excluded. After further screening, 144 papers satisfied the inclusion criteria and were considered for qualitative analysis, of which 91 were finalized for quantitative analysis. We identified 78 papers with in vitro, 15 with in vivo and 19 with compound studies, whereas the IC_50_ value was measured only in six studies.

All articles pertaining to echinococcosis and therapeutic herbal plants were chosen. In addition, the reference lists of all related papers were examined to ensure that no significant data was missing. The search was conducted in the English language. Repetitive publications and papers with a poor technique were all removed from consideration.

### 2.3. Study Selection

Initially, three investigators (MAA, SK and RMAA) retrieved the articles and assessed their titles and abstracts for the eligibility criteria. Then, the relevant full text published articles were reviewed by three investigators (WQ, MS and NYF). In the case of any controversy, two more investigators (LL and HBY) were invited to discuss the article. Information including the species of plant used, part(s) used, extraction method, phytochemical component, concentration (mg/mL), exposure time (min), scolicidal efficacy (%), and year of publication of the work were considered in the selection process.

### 2.4. Data Extraction

The following details were gathered: initial author, parasite species, herbal plant, in vitro study, in vivo experiment, dose rate, efficacy, exposure time, part used, year of publication, and half-maximal inhibitory concentration (IC_50_) value.

### 2.5. Statistical Analysis

Descriptive analysis was applied to review the scolicidal activity of herbal plants. The dose rate, time duration, efficacy and part of the plant used were the minimum requirements to conduct the analysis. All of the data were extracted and arranged using an Excel spreadsheet (Office 365 Version 2019; Microsoft Corporation, WA, USA). The research papers were described in the form of tables. The subgroup analysis was conducted using MedCalc software version 20.014. The mean of the IC_50_ value with a 95% CI was calculated using the random effect model and represented in the form of a forest plot. Cochran’s Q and I^2^ statistics were estimated to access the heterogenicity. The graphs were made with Datawrapper (https://www.datawrapper.de/, accessed on 19 March 2022).

## 3. Results

Out of 153 research articles, searched from 2000 to 2021, we found 91 publications (78 in vitro and 15 in vivo) that matched our eligibility criteria and were included in the present systematic review. Our systematic review excluded unpublished data, duplicated publications, the same data published elsewhere, and inaccessible data. In total, ninety-one different categories of data were retrieved, retaining information on the plant species, extraction method, plant part, concentration, exposure time, efficacy, and species of *Echinococcus*. All plant extracts were tested in vitro as well as *in vivo*. Limited reports also possessing information on the IC_50_ value were also retained. We attempted to summarize several pieces of research in the list of anti-*Echinococcus* herbs and natural products. The majority of echinococcicidal compounds investigated came from natural sources. Characters such as the plant part, extraction method, methodology being in vitro or in vivo, active components, toxicity, and IC_50_ value were subjected to subgroup analysis.

**Figure 2:**
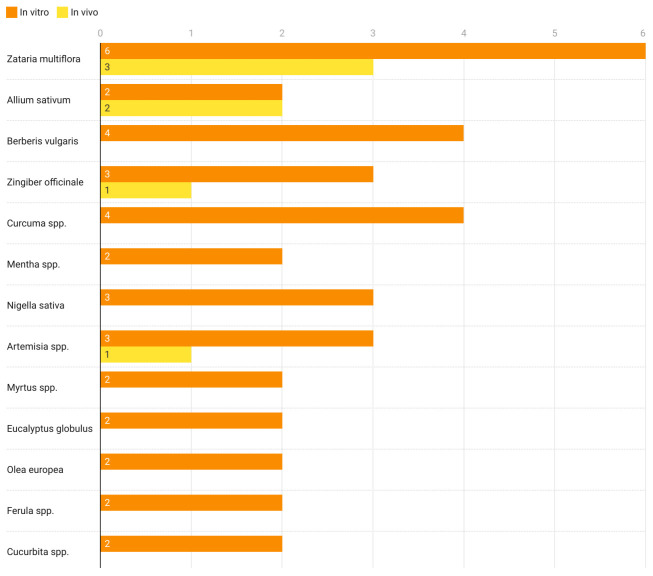
Plant-wise comparison of in vitro and in vivo studies.

**Figure 3:**
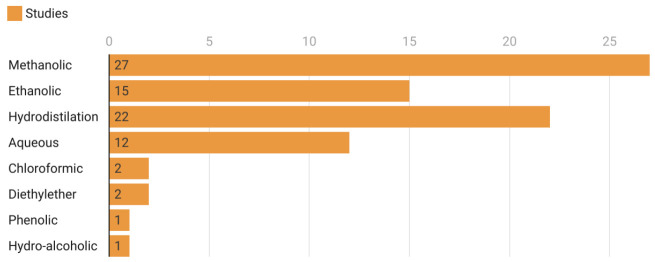
Comparison of extraction methods used in 91 studies.

A total of 63 plant species used against the scoleces of hydatid cysts (in vitro and in vivo) were included in this systematic review, among which, a few were used more frequently (Figure 2) and the methanolic extraction was the most common method employed (Figure 3).

### 3.1. Parts of Medicinal Plants Used as Scolicidal Agents

**Figure 4:**
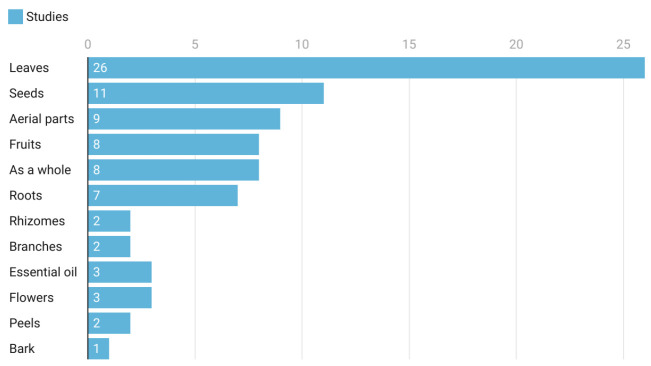
Comparison of parts of medicinal plants used in this systematic review.

The most commonly used part was discovered to be the leaves, which were employed in 25.6 percent of the research, followed by seeds (15.1 percent), aerial parts (13.9 percent), fruit (10.4 percent), and roots (5.8 percent) (Figure 4). Other parts were comprised of rhizomes, branches, essential oil, flowers, peels and bark. A single plant species was used in all formulations against *Echinococcus* scoleces except one report in which two plant fruits (grape and apple) were used.

### 3.2. In Vitro Activity of Medicinal Plants against Protoscoleces

**Figure 5:**
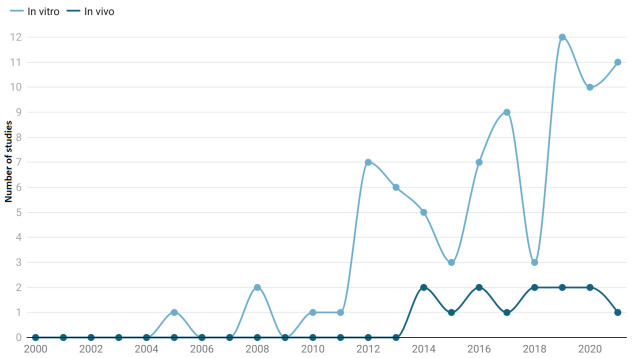
Year-wise comparison of in vitro and in vivo studies.

Our systematic review revealed a total of 58 species that were used in the in vitro studies as an echinococcicidal agent (Figure 5). *Zataria multiflora* extract was used most commonly to kill the protoscoleces, followed by *Nigella sativa*, *Berberis vulgaris*, *Zingiber officinale*, and *Allium sativum* (Table 1). Herbs among plant forms, methanolic extract among extraction, and leaves among herbs, were extensively used in the in vitro studies. Plants such as *Zataria multiflora*, *Ferula assafoetida* and *Berberis vulgaris* were found to have a higher efficacy in the in vitro experiments. *Zataria multiflora* killed all scoleces at a concentration of 1 mg/mL in 5 min. *Ferula assafoetida* and *Berberis vulgaris* were found to have 100% efficacy at concentrations of 60 μg/mL and 2 mg/mL for 10 min.

### 3.3. In Vivo Activity of Medicinal Plants against Scoleces

Several medicinal herbs and pure phytochemicals are being studied for their anti-*E. granulosus* preventative and therapeutic properties, in order to develop novel CE treatments with fewer or milder adverse effects. Two plant species, *Zataria multiflora* and *Allium sativum*, were potentially employed in the in vivo studies for their validation against *E. granulosus* protoscoleces in this study. Leaf extracts, peels and other parts were tested to validate the in vivo anti-*Echinococcus* activity (Table 2).

### 3.4. Active Phytochemical Compounds against Scoleces

A total 17 phytochemical compounds were collected from different herbal plants against *E. granulosus* mentioned in this systematic review. Out of these active compounds, seven phytochemicals, comprising of flavonoids, thymol, carvacrol, phenol, alkaloids, gallic acid and polyphenol, were commonly used both in the in vitro as well as in vivo studies. Flavonoid, thymol and carvacrol showed the highest scolicidal activity at concentrations of 0.49, 1, and 10 mg/mL for 0.5, 5. and 10 min in the in vitro studies (Table 1). Flavonoids and thymol also revealed significant scolicidal activity in the in vivo studies (Table 2).

### 3.5. Herbal Scolicidal Plants Toxicity

Tests on herbal plant extracts were conducted to evaluate their toxicity levels. The most appropriate scolicidal herbs are those that do not have any toxicity and kill all scoleces with a minimum concentration in a very short period of time. Results of this systematic review revealed that only one species (*Zataria multiflora)* was tested for toxicity and it proved to be safe, and no toxicity was observed when it was used in pregnant mice [97,99].

### 3.6. IC_50_ Value Analysis

**Figure 6:**
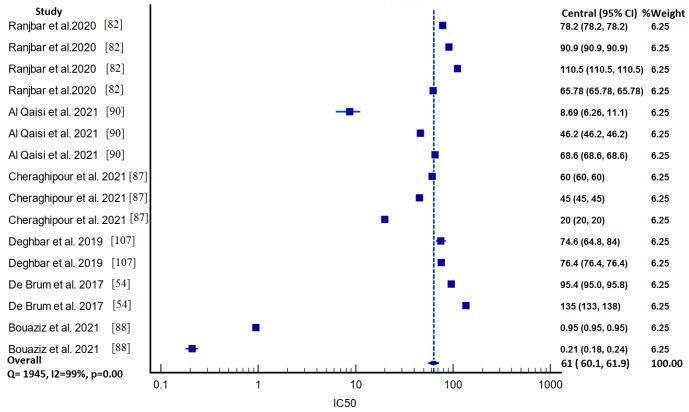
Forest plot indicating pooled IC_50_ value along with 95% CI.

Out of 91 articles, the IC_50_ value was identified in only six studies. When analysis was performed to identify the pooled value using Medcalc software, the Q statistic value was found to be very high (Q 1945, DF 16, I-square 99%, *p* < 0.001), indicating that there was a lot of variation between the investigations. The pooled mean of IC 50 was 61 according to the random effect model (95% CI 60–61.9), as shown in Figure 6. We could not compare our results with others because the selection criteria and randomization standard differed between the studies.

A comprehensive comparison of the variation in IC_50_ could not be made because the extraction methods used in all the studies were different (Table 3). Furthermore, the parts of the plants used in the subject studies also varied. Additionally, the duration of exposure in these studies was also different.

## 4. Discussion

For a long time, herbal medicines have occupied a pivotal position in complementary and alternative medicine throughout the world [112]. According to the World Health Organization (WHO), more than 70% of the world’s population rely on folk remedies for the treatment of some of their health care issues [75].

The global trend in research is facing a shift as it is more focused towards the exploration of new medicines rather than the cultivation of plant species that have a therapeutic significance [113]. Until the advent of the 18th century, the therapeutic properties of many plants were known, but little knowledge existed about the active compounds [114]. Despite the advantages of herbal medicines, traditional medicine may pose serious drawbacks, such as the use of medicinal plants without taking sanitary conditions into account or considering the possible harmful effects on health [115]. Furthermore, insufficient research methodologies lead to the proliferation of products, giving rise to false perspectives [116].

In this study, a total of 91 plants were recorded therapeutically and examined for their efficacy against the protoscoleces of *E. granulosus* in this literature review. In comparison to shrubs and trees, herbs were discovered to be the most commonly used type of plants against helminth infections. The dominance of herbs over the other forms of plants can be linked to their widespread availability and strong efficacy against a variety of diseases, when compared to shrubs and trees [117]. Herbs are commonly applied in natural medicines all over the world and they contain a huge number of active ingredients, which accounts for their great efficacy and keeps them as the first choice of scientists and as alternative medicines [118]. When comparing trees with herbs and shrubs, our findings revealed that trees are the plant-life type that is used least often, which might be attributed to concerns related to biodiversity and ecological effects. Due to overharvesting, some tree species have been designated as endangered. In these circumstances, current procedures such as cloning, callus cultivation, natural cultivation, genetically modified cultures, and multiplication should be utilized to obtain chemical elements of therapeutic importance and to solve the resource imbalance [119].

It was found that leaves were used more frequently, compared with other parts, in medicinal confirmation of therapeutic activity against *E. granulosus*. Herbalists favor leaves because they seek a constant raw material supply [120]. Furthermore, leaves may be easily picked without causing significant damage to plants, which may explain why leaves are the most commonly used plant element [121]. According to reports, leaves contain several bioactive constituents that have a variety of medical properties [118]. On the other hand, Albuquerque (2006) observed that such extensive usage of leaves in herbal medicine might potentially limit plant development, resulting in fewer plant recipes [122]. Flavonoids, saponins, tannins, and other phytochemical substances are found in plant seeds, and it appears that these phytochemical components are important in the bioactivity of medicinal plants [123]. Roots serve as nutrient storage sites for plants, which may indicate why they are widely utilized in herbal medicine [124]. Root harvest, on the other hand, frequently results in the plant’s mortality and poses a serious risk to conservation [125].

Moreover, collecting entire plants for anti-parasitic activity assessment is controversial from a conservation standpoint [126]. The frequent usage of essential oils and methanolic extracts highlights the importance of solvents in extracting bioactive chemicals from various plants and their components. Methanol is particularly good at extracting bioactive chemicals from plants due to its polarity [127]. This might be one of the reasons for the widespread use of this solvent in herbal preparations. However, on the other hand, essential oils have been shown to have anthelmintic properties [128]. Furthermore, they possess terpenes that disrupt parasitic biochemical and physiological processes. Despite the fact that combinations of multiple plants and their preparations are often more efficacious than a single extract, only one polyherbal preparation was identified against the protoscoleces of *E. granulosus* [80]. This is an evident research gap and polyherbal preparations should be examined in future studies. In vitro testing of the medicinal herbs described demonstrates their efficacy against the protoscoleces of *E. granulosus*, and the leaves were dominant across plant components and herbs were prominent across plant life forms.

The results show that most of the research was concentrated on the in vitro rather than in vivo assessment of plants against *E. granulosus* protoscoleces. This might be due to the fact that in vitro plant testing is less expensive, takes less time, and produces rapid results, allowing for large-scale plant testing. Furthermore, these studies directly examined the influence of anthelmintic plants on parasite hatching, maturation, and movement, without compromising the host’s basic physiological functions [129]. Another benefit of in vitro investigations is that, if credible findings are obtained, the isolate may be tested in vivo [130]. However, herbal extracts that are active in vitro may or may not be successful in vivo [131]. This type of difference in activity in the evaluation of new antiparasitic substances is fairly common and it can be linked to a variety of factors, including the bioavailability, intrinsic pharmaceutics of the compound evaluated, the possible damage, or poor solubility of the compounds in the rumen of ruminants and parasite protective mechanisms [132]. This constraint emphasizes the necessity of pharmacokinetic and pharmacodynamic investigations in the discovery of potential novel anthelminthic drugs against *E. granulosus* for industrial use.

*Zataria multiflora* essential oils showed a remarkable anti-hydatid action with short exposure times [44]. This powerful activity of *Zataria multiflora* oil is likely related to the significant phenolic monoterpene ingredients. The antimicrobial action of phenolic monoterpenes may be due to their innate hydrophobic nature and the presence of a hydroxyl group; hence, these chemicals damage cells by penetrating the cell membrane [133]. Although the mode of action of phenolic monoterpenes versus protoscoleces has yet to be determined, research on other eukaryotic cells has shown that phenolic monoterpenoids primarily act on plasma and mitochondrial membranes, causing cell death. They penetrate through the membrane, damaging the lipid bilayer and, as a result, changing the cell permeability, which increases ion leakage and lowers the membrane electric potential. This change in the plasma membrane electric potential probably causes leaking of ATP, amino acids, proteins, and electrolytes, particularly potassium and calcium, resulting in membrane damage and cell death [134]. Furthermore, changing the molecular structure of the mitochondrial membrane causes protein, radical, calcium, and cytochrome c leaks, leading to apoptosis [135].

In terms of exposure time and efficacy, *Ferula assafoetida* essential oils were more successful than the other plants mentioned in this systematic review. The essential oils from these herbs, which contain disulfide compounds, were tested against several eukaryotic malignant cells for their ability to cause cell death, which depicts its scolicidal action [136].

The pharmacological mechanism of action of most anti-hydatid plants are unknown, and more research is needed in this sector to provide complete information on the scientific basis of indigenous medicinal plants, in order to produce a new scolicidal medicine.

In vivo tests are necessary to analyze the pharmacokinetics/pharmacodynamics of the target extract, as well as the host immune reaction to the target extract. In vivo investigations provide a variety of advantages, but they also have certain drawbacks. In vivo investigations are clearer and more explicit than in vitro research, but they are more time consuming, expensive, and difficult to replicate, due to pharmacodynamic interactions within the host [137]. In conclusion, both strategies have critical tasks to perform, and one does not preclude the other [138].

In vivo, *Zataria multiflora* was applied to fight protoscoleces. The utilization of this plant parts (leaves) was consistent with the indigenous medicinal practice, which demonstrates the current scientific beliefs in ethnomedicine. The mouse is the most widely utilized in in vivo echinococcosis research. This may be due to the great resemblance of its genome to that of humans. Furthermore, because of its short generation time, tiny size, and ease of breeding, it is a cost-effective tool for in vivo investigations for obtaining functional information on human health and disorders [139]. We reported the isolation and evaluation of eight compounds for scolicidal activity versus protoscoleces. Primary and secondary metabolites are physiologically active chemicals found in plants. Primary components include chlorophyll, proteins, and carbohydrates, whereas secondary compounds include flavonoids, alkaloids, terpenoids, and phenols [140]. These many bioactive chemicals interact synergistically to create a therapeutic action [141]. Furthermore, the high activity of plant extracts might be due to a greater therapeutic connection between the distinct main components, which can connect with many molecular targets at different phases of parasite growth [142].

With the advancements occurring in the field of medicine, researchers are more directed towards investigating/determining the active compounds of plants to treat certain diseases [113]. Most of the studies conducted to evaluate the protoscolicidal activity of different herbs during the last two decades have investigated their in-vitro activity. A few studies were done in-vivo in animal models. In humans, hydatid cyst surgery and the infiltration of protoscolicidal agents has serious side effects of spilling over, that may lead to other complications. More attention is required to be paid to the toxicity of these drugs, as well as the search for other suitable alternative drugs [143]. An appropriate protoscolicidal agent should show its activity at lower doses with high efficiency in a shorter period of time. Furthermore, a good protoscolicidal agent is considered to be steady in the cystic contents and must possess the least toxicity [144]. For a promising protoscolicidal agent, additional in-vivo studies are recommended to prove them a good scolicidal agent in a clinical setting.

In this study, we found that there is a lack of information on the toxicology and pharmacology of several medicinal plants and their components. Additional study is needed to determine the toxicity and pharmacology of the herbs and chemicals with potential scolicidal action.

## 5. Conclusions

In conclusion, the current study found that a variety of herbal extracts have impacts on *Echinococcus* stages, as well as anti-*Echinococcus* abilities in vitro and in vivo. As a result, extracts might potentially be used instead of pharmaceutical medications. However, most of the authors reported that their researched plants had yielded positive results, although their studies contained flaws that influenced the results of their findings. Some of the flaws in this research are detailed, such as the absence of randomized double-blind clinical trials in all human investigations. In addition, several of the studies were conducted in vitro rather than in vivo. The majority of the data published came from animal models but have not been trialed on humans. Herbal therapy has presented a vast and positive vision of novel, safe, and potent anti-*Echinococcus* herbal medicines, according to all published research. To confirm their actions, it is necessary to generalize the data gained from in vitro and in vivo investigations on the effectiveness of plant extractions and metabolites against *Echinococcus* species. The current thorough examination of herbal plants’ anti-*Echinococcus* activity, as well as their toxic effects and mode of action, has the most potential for confirming their therapeutic efficacy against echinococcosis. Overall, the systematic review gives valuable information regarding natural medicines with anti-*Echinococcus* activity, which will be used in clinical and experimental trials, as well as plant combination treatment research. As a result, additional clinical trials are needed to determine whether herbal plant treatment is beneficial and safe. It is important to identify their active ingredients, as well as any potential adverse consequences, in order to develop well-tolerated and safe therapies against echinococcosis.

## Figures and Tables

**Table 1:** In vitro efficacy of medicinal plants against protoscoleces of *Echinococcus granulosus*.

Year	Botanical Name (Common Name)	Extraction Method	Part Used	Phytochemical Component	Concentration (mg/mL)	Exposure Time (min)	Scolicidal Efficacy (%)	References
2005	*Peganum harmala* (Syrian rue)	Ethanolic	Seeds	N/A	62.5	2880	100	[20]
2008	*Allium Sativum* (Garlic)	Ethanolic/chloroform	Garlic cloves	Silver nitrate	200	15	70	[21]
2008	*Coriandrum sativum* (Coriander)	Hydrochloric acid + diethyl ether	Seeds	Phenols	750	10,080	100	[22]
2010	*Allium sativum*(Garlic)	Methanolic	Garlic cloves	Mannitol	50	10	100	[23]
2011	*Zingiber officinale* (Ginger)	Methanolic	Rhizome	N/A	100	30	100	[24]
2012	*Mentha* spp.	Hydrodistillation	Leaves	Isomenthol	0.005	25,920	100	[25]
2012	*Corylus* spp.	Hydro-alcoholic	Seeds	N/A	50	20	98	[26]
2012	*Olea europaea* (Olive)	Aqueous	Leaves	N/A	1	120	96.7	[27]
2012	*Zataria multiflora* (Shirazi thyme)	Methanolic	Leaves	Carvacrol and Thymol	25	1	100	[28]
2012	*Rhus coriaria* (Sumac)	Methanolic	As a whole	N/A	30	20	98.89	[29]
2012	*Trachyspermum ammi* (Ajowan)	Hydrodistillation	Fruits	Thymol	5	10	100	[30]
2012	*Satureja khuzistanica* (Jamzad)	Hydrodistillation	Leaves and flowers	Carvacrol	5	60	100	[31]
2013	*Hymenocarter longiflorus* (Lamiaceae)	Methanolic	Aerial parts	Monoterpene hydrocarbons (2.25%), Hydrocarbons (17.44%), Oxygenated monoterpene (19.27%)	0.0125	2880	100	[32]
2013	*Ferula assafoetida* (Assafoetida)	Hydrodistillation	Latex	E-1-propenyl-sec-butyl Disulfide (62.7%), β-ocimene (21.7%) and β-pinene (5%)	0.06	10	100	[33]
2013	*Ocimum bacilicum* (Sweet basil)	Methanolic	Leaves	N/A	100	60	24.10	[34]
2013	*Berberis vulgaris* (Barberry)	Aqueous	Fruit	N/A	4	30	100	[35]
2013	Pestalotiopsis spp.	Methanolic	Leaves, stems and roots	N/A	30	30	92	[36]
2013	*Mallotus philippinensis* (Kamala Tree)	Methanolic	Fruit	N/A	20	120	100	[37]
2014	*Thymus vulgaris* (Garden thyme)	Hydrodistillation	Leaves	Thymol	0.5	103,680	100	[38]
2014	*Nigella sativa* (Black Cumin)	Methanolic	Seeds	Thymoquinone	50	30	100	[39]
2014	*Berberis vulgaris* (Barberry)	Methanolic	Root	Berberine	2	10	100	[40]
2014	*Nigella sativa* (Black Cumin)	Hydrodistillation	Seeds	Thymoquinone	10	10	100	[41]
2015	*Teucrium polium* (Felty germander)	Ethanolic	Flowers	N/A	100	50	100	[42]
2015	*Zingiber officinale* (Ginger)	Methanolic	Root	N/A	100	40	100	[43]
2015	*Zataria multiflora* (Shirazi thyme)	Diethyl ether	Essential oil	Thymol (66.9%), Carvacrol (15.2%), Carvone (7.3%), Neo-dihydrocarveol (2%), and 1,8-Cineole (1.6%)	1	5	100	[44]
2016	*Bunium persicum* (Black Caraway)	Hydrodistillation	Seeds	g-terpinene (46.1%), Cuminaldehyde (15.5%), r-Cymene (6.7%), and Limonene (5.9%)	0.0125	10	100	[45]
2016	*Pistacia khinjuk* (Khiniuk)	Methanolic	Fruits	Terpenoids, Flavonoids, and Tannins	100	10	100	[46]
2016	*Salvadora persica* (Miswak)	Ethanolic	Root	Indole alkaloids, Flavonoids, Tropaedoin, Triterpenes, Phytosterols, and Isothiocyanates	50	10	100	[47]
2016	*Zataria multiflora* (Shirazi thyme)	Methanolic	Leaves	Carvacrol and Thymol	10	10	100	[48]
2016	*Myrtus communis* (True myrtle)	Hydrodistillation	Leaves	α-pinene (24.7%), 1,8-Cineole (19.6%), and Linalool (12.6%)	0.1	5	100	[49]
2016	*Nectaroscordum tripedale* (Sicilian Honey Garlic)	Ethanolic	Leaves	Terpenoids, Flavonoids, Tannins and Fatty acids	50	10	100	[50]
2016	*Zingiber officinale* (Ginger)	Aqueous	As a whole	[6]-gingerol	100	1440	100	[51]
2017	*Curcuma longa* (Turmeric)	Ethanolic	As a whole	N/A	30	30	100	[52]
2017	Bunium Persicum (Black Caraway)	Hydrodistillation	Seeds	Β-terpinene (28%)	15	10	100	[53]
2017	*Poikilacanthus glandulosus* (Ariza)	Ethanolic	Branches	Polyphenols and Flavonoids	0.01	15	100	[54]
2017	*Ephedra (Mormon tea)*	Methanolic	Root, stem and leave	N/A	1	60	99.09	[55]
2017	*Artemisia sieberi* (Wormwood)	Hydrodistillation	Aerial parts	Alpha-Thujone (31.5%)	0.005	120	99.30	[56]
2017	*Zataria multiflora* (Shirazi thyme)	Hydrodistillation	Aerial parts	Thymol (41.8%), Carvacrol (28.8%), and p-Cymene (8.4%)	0.1	10	100	[57]
2017	*Blepharocalyx salicifolius* (Kunth)	Aqueous	Leaves	Gallic acid and Rutin	200	5	100	[58]
2017	*Cinnamomum zeylanicum* (Cinnamon)	Hydrodistillation	Bark	Cinnamaldehyde (91.8%), Metoxicinamate (1.57%), and α pinene (1.25%)	0.05	5	100	[59]
2017	*Melaleuca alternifolia* (Tea tree)	N/A	Tree oil	Terpinen-4-ol (35.4%), α-Terpinene (11%), γ-Terpinene (20.4%) and 1,8-Cineole (3.4%)	20	5	90	[60]
2018	*Berberis vulgaris* (Barberry)	Methanolic	Aerial parts	N/A	100	40	100	[61]
2018	*Artemisia* (Wormwood)	Methanolic	NA	N/A	100	15	97.24	[62]
2018	*Cucurbita maxima* (Pumpkin)	Methanolic	Seeds	Spinasterol	50	60	100	[63]
2019	*Artemisia sieberi* (Wormwood)	Aqueous	As a whole	N/A	50	20	100	[64]
2019	*Myrtus communis* (True myrtle)	Methanolic	Leaves	N/A	100	20	100	[65]
2019	*Eucalyptus globulus* (Bluegum)	NA	Leaves	Eucalyptol (79.32%)	5	3	100	[66]
2019	*Olea europaea* (Olive)	Ethanolic	Leaves	N/A	150	25	100	[67]
2019	*Berberis vulgaris* (Barberry)	Ethanolic	Aerial parts	Flavonoids, Alkaloids and Saponins	50	30	97.92	[68]
2019	*Citrullus colocynthis* (Colocynth)	Methanolic	Fruits	N/A	16	120	100	[69]
2019	*Eucalyptus globules* (Bluegum)	Aqueous	Leaf	Eucalyptol (79.32%)	10	5760	94	[70]
2019	*Allium sativum* (Garlic)	Chloroformic	Fresh garlic	N/A	200	1	100	[71]
2019	*Satureja hortensis* (Summer savory)	Aqueous	Aerial parts	Carvacrol and γ-terpinene	1	20	100	[72]
2019	Zingiber officinale (Ginger)	Ethanolic	Rhizomes sheets	N/A	200	30	100	[73]
2019	*Punica granatum* (Pomegranate)	Alcoholic	Stem and root	N/A	9	1440	100	[74]
2019	*Curcuma longa* (Turmeric)	Hydrodistillation	Rhizome	α-turmerone (27.1%) β- turmerone (21.8%), l-phellandrene (8.8%), and ρ-cymene (5.4%)	0.1	5	100	[75]
2019	*Pelargonium roseum*	Hydrodistillation	Leaves	N/A	0.05	60	100	[76]
2020	*Lepidium sativum* (Garden cress)	Aqueous	Leaves	N/A	100	15	100	[77]
2020	*Taxus baccata* (Common yew)	Hydroalcoholic	As a whole	Octane (13.36%), 4-methoxycarbonyl 3,5-diphenyl-1 (8.30%), and 9,12,15-Octadecatrienoic acid (10.75%)	150	60	66.60	[78]
2020	*Cucurbita moschata* (Pumpkin)	Hydroalcoholic	Seeds	N/A	1	60	16	[79]
2020	Grape + apple vinegar	N/A	As a whole	N/A	5	5	100	[80]
2020	*Cannabis sativa* (Hemp)	N/A	Aerial parts	N/A	0.01	10	26.08	[81]
2020	*Mentha* species (Lamiaceae)	Methanolic	Aerial parts	Phenolic, Flavonoid and Flavonol contents	200	10	99.54	[82]
2020	*Curcuma zadoaria* (White turmeric)	Hydrodistillation	Rhizome	Pentadecane (29.6%), Delta-3-Carene (14.7%), and Cis-Cinnamic Acid (8.4%)	0.15	7	100	[83]
2020	*Hibiscus sabdariffa* (Roselle)	Aqueous	As a whole	N/A	2	5	100	[84]
2020	*Ziziphora tenuior* (Mint)	Ethanolic	Shoots	Thymol	100	240	40.25	[85]
2021	*Capparis Spinosa* (Caper)	Methanolic	Fruit	Flavonoids, Tannins, Terpenoids, Glycosides and Alkaloids	300	20	100	[86]
2021	*Piper longum* (Long pepper)	Methanolic	Fruits	Phenolics, Flavonoids, Alkaloids, Tannins, Terpenoids, and Glycoside	100	60	100	[87]
2021	*Atriplex halimus* (Orache)	Aqueous	Leaves	Phenolic and Flavonoids	60	120	99.36	[88]
2021	Sideritis perfoliate (Ironwort)	Methanolic	Leaves and flowers	Fumaric acid (260.13 mg/L), Syringic acid (27.92 mg/L) and Caffeic acid (26.84 mg/L), and a Flavonoid, luteolin (11.23 mg/L)	25	60	100	[89]
2021	*Ruta graveolens* (Common rue)	Methanolic	Aerial parts	Phenolic (25.53%), Flavonoids (6.6%) and Tannins (8.0%)	40	720	100	[90]
2021	*Saussurea costus* (Costus)	Ethanolic	Root	N/A	250	60	100	[91]
2021	*Zataria* spp. (Satar)	Hydrodistillation	Leaves	Carvacrol and Thymol	100	1	100	[92]
2021	*Allium noeanum* (Reut)	Ethanolic	Leaves	Flavonoid	0.49	0.5	100	[93]
2021	*Ferula macrecolea* (Koma)	Hydrodistillation	Leaves	Terpinolene (77.72%), n-Nonanal (4.47%), and Linalool (4.35%)	0.3	10	100	[94]
2021	*Cassia fistula* (Golden shower)	Ethanolic	Fruits	N/A	100	60	67.74	[95]
2021	*Silybum marianum* (Milk thistle)	Ethanolic	Seeds	Silydianin (14.41%), Isosilybin A (10.50%), and Silychristin (10.46%)	0.5	60	77	[96]

N/A = Not available.

**Table 2:** In vivo efficacy of medicinal plants against protoscoleces of *E. granulosus*.

Year	Botanical Name (Common Name)	Extraction Method	Part Used	Phytochemical Component	Experimental Animal	Concentration (mg/mL)	Exposure Time (min)	Scolicidal Efficacy (%)	References
2014	*Zataria multiflora* (Shirazi thyme)	Diethyl ether	Aerial parts	Gallic acid (1.1618 mg/g), Catechin(2.808 mg/g), Caffeic acid (5.531 mg/g), and Quercetin (9.961 mg/g)	Mice	0.04	43,200	Significant	[97]
2014	*Zingiber officinale* (Ginger)	Ethanolic	As a whole	N/A	Mice	150	60	100	[98]
2014	*Zataria multiflora* (Shirazi thyme)	Methanolic	Leaves	Thymol (66.9%), Carvacrol (15.2%), and Carvone (7.3%)	Mice	8	43,200	100	[99]
2015	*Artemisia Herba-alba* (Wormwood)	Ethanolic	Leaves and flowers	Alkaloids, Phenols	Mice	0.28	1440	55.17	[100]
2016	*Pistacia vera* (Pistachio)	Hydrodistillation	Branch	Essential oil	Mice	200	10	100	[101]
2016	*Punica granatum* (Pomegranate)	Aqueous	Peels	N/A	Mice	16	2880	100	[102]
2017	*Zataria multiflora* (Shirazi thyme)	Hydrodistillation	Essential oil	Thymol	Mice	2	10	100	[103]
2018	*Allium sativum* (Garlic)	Methanolic	Garlic cloves	1% Alliin	Mice	80	43,200	Significant	[104]
2018	*Sophora moorcroftiana*	N/A	Seeds	N/A	Mice	0.25	60,480	76.1	[105]
2019	*Punica granatum* (Pomegranate)	Aqueous	Peel	N/A	Mice	0.65	86,400	66.7	[106]
2019	*Algerian propolis* (Propolis)	Ethanolic	N/A	Polyphenol, Flavonoid	Mice	25	10	100	[107]
2020	*Nigella sativa* (Black cumin)	Ionotropic gelation technique	Seed	N/A	Mice	1.14	86,400	100	[108]
2020	*Zataria multiflora*	Essential oil and oleic acid	Essential oil	N/A	Mice	20	10	100	[109]
2021	*Allium sativum* (Garlic)	Methanolic	N/A	N/A	Mice	50	10	100	[110]
2021	*Annona squamosa* (Sugar apple)	Alcoholic	Leaves	N/A	Rats	100	2880	100	[111]

N/A= Not available.

**Table 3:** Studies with IC_50_ having effects against protoscoleces.

Plant	Part of Plant Used	Extraction Method	IC_50_
*Piper longum*	Dry fruits	Methanolic extract	20 mg/mL for 60 min
*Ruta graveolens* L.	Stems and leaves	Methanolic Extract	40 mg/mL for 120 min
*Atriplex halimus*	Leaves	Aqueous extract	40 mg/mL for 90 min
*Algerian propolis*	Buds of poplar and cone-bearing trees	Ethanolic extract	74.65 ± 9.79 µg/mL
Iranian *Mentha*	Stems, leaves, and fruits	Physiological serum	200 mg/mL 30 min

## Data Availability

Data is contained within the article or Supplementary Materials.

## Supplementary Materials

The following supporting information can be downloaded at: https://www.mdpi.com/article/10.3390/life12050676/s1, Table S1: PRISMA 2009 checklist. [19]
